# Intraocular Pressure Response in the Untreated Contralateral Eye After Selective Laser Trabeculoplasty

**DOI:** 10.7759/cureus.67537

**Published:** 2024-08-22

**Authors:** Jason Dossantos, Tyler Muser, Devin Hill, Stephen Lesche, Aseef Ahmed, David Belyea

**Affiliations:** 1 Department of Ophthalmology, George Washington University School of Medicine and Health Sciences, Washington, USA

**Keywords:** contralateral effect, glaucoma medication, contralateral eye, selective laser trabeculoplasty, primary open angle glaucoma

## Abstract

Aim: To examine the impact of selective laser trabeculoplasty (SLT) on intraocular pressure (IOP) in the untreated contralateral eye within 12 months after the procedure. Methods: A retrospective chart review was conducted on patients with primary open-angle, normal-tension, pigmentary, or pseudoexfoliation glaucoma who received 360-degree SLT at George Washington University. Exclusion criteria included prior or subsequent laser or glaucoma surgery within 12 months of SLT, other glaucoma types, or corticosteroid use during follow-up. Primary outcomes were IOP and medication reduction, and SLT success, defined as reducing IOP by ≥20% without additional IOP-lowering procedures or medications. Follow-up occurred at six weeks, six months, and 12 months. Demographic and clinical data were analyzed using ANOVA, paired t-tests, and chi-squared tests.

Results: A total of 125 patients were included, representing a range of backgrounds: African American (57.6%), Caucasian (31.2%), Asian (5.6%), and Hispanic/Latino (4%), and 1.6% did not report their background. Significant reductions in mean IOP and medication numbers were observed in the contralateral eye at six weeks and six months (p<0.05) but not at 12 months. The contralateral eye success rates were 24% at six weeks and six months and 20.8% at 12 months. The contralateral eye was more likely to achieve success if the ipsilateral eye was successful at six weeks (odds ratio (95% confidence interval): 5.05 (1.89-13.48)), six months (16.1 (4.56-57.17)), and 12 months (5.94 (2.07-17.04)) (p<0.001 for all).

Conclusion: First-time SLT results in statistically significant IOP and medication reductions in the contralateral eye at six weeks and six months. The contralateral eye was 5.05-16.1 times more likely to achieve success if the ipsilateral eye was successful within 12 months.

## Introduction

Selective laser trabeculoplasty (SLT) is a widely used method for lowering intraocular pressure (IOP) in glaucoma, both as a primary and supplementary treatment option [1]. First introduced by Latina et al. in 1995, SLT uses a 532 nm Q-switched, frequency-doubled Nd:YAG laser to selectively target the pigmented cells of the trabecular meshwork [2]. The laser induces the release of cytokines, which bind to the endothelial cells of Schlemm's canal, enhancing aqueous humor outflow and reducing IOP. Multiple studies have demonstrated the effectiveness and safety of SLT in treating various forms of glaucoma, providing an essential alternative to traditional treatments that often involve more invasive methods [3-5].

While the primary benefits of SLT are well recognized, observations of its effects on untreated (contralateral) eyes have sparked significant interest. Initial observations by Latina et al. revealed a modest IOP reduction in the untreated eye, hypothesized to result from systemic absorption of cytokines or other mediators released during laser treatment [6]. This short-term bilateral effect was subsequently supported by several studies, which proposed a systemic inflammatory mechanism, with varying duration from six weeks to three years [7-11]. Hirabayashi et al. further revealed that untreated eyes were six times more likely to meet their success criteria (≥20% IOP reduction or ≥1 medication reduction without further IOP-lowering interventions) at six months if the treated (ipsilateral) eye was also successful [11]. However, contrasting findings from Gulati et al. indicated no significant IOP changes in the untreated eye [12]. Moreover, several studies lacked diversity in their populations and excluded patients with increases in medication during follow-up, thus limiting the external validity of the findings and highlighting potential variability in patient response and study methods [7-12].

This study seeks to evaluate the bilateral effects of SLT on IOP and medication reduction over 12 months across a broader, more diverse cohort. By investigating the success rate of both the ipsilateral and contralateral eye after SLT, this study also seeks to clarify the extent to which the response in the ipsilateral eye can predict successful outcomes in the contralateral eye.

## Materials and methods

A retrospective review of medical records was conducted on 1,421 glaucoma patients who underwent 360° SLT between January 1, 2008, and January 1, 2020, at the George Washington University Department of Ophthalmology. This study included patients diagnosed with primary open-angle, pseudoexfoliation, pigmentary, or normal-tension glaucoma. IOP was measured by an ophthalmologist using Goldmann applanation tonometry, and medication adjustments were made by the treating physician based on clinical judgment and patient-specific factors. Commonly used medications for reducing IOP included beta-blockers such as timolol; alpha agonists such as brimonidine; prostaglandin analogs such as latanoprost, travoprost, and bimatoprost; and carbonic anhydrase inhibitors such as dorzolamide. In this study, these medications were typically administered as follows: timolol 0.25%-0.5% twice daily, brimonidine 0.1%-0.15% twice daily, latanoprost 0.005% once daily, travoprost 0.004% once daily, bimatoprost 0.01%-0.03% once daily, and dorzolamide 2% twice daily. Ethical approval for the study was obtained from the Institutional Review Board of the George Washington University School of Medicine and Health Sciences. Due to its retrospective design, the study was exempt from the requirement for informed consent. All procedures and data collection adhered to the Health Insurance Portability and Accountability Act (HIPAA) regulations and the principles of the Declaration of Helsinki.

Selection criteria

**Figure 1:**
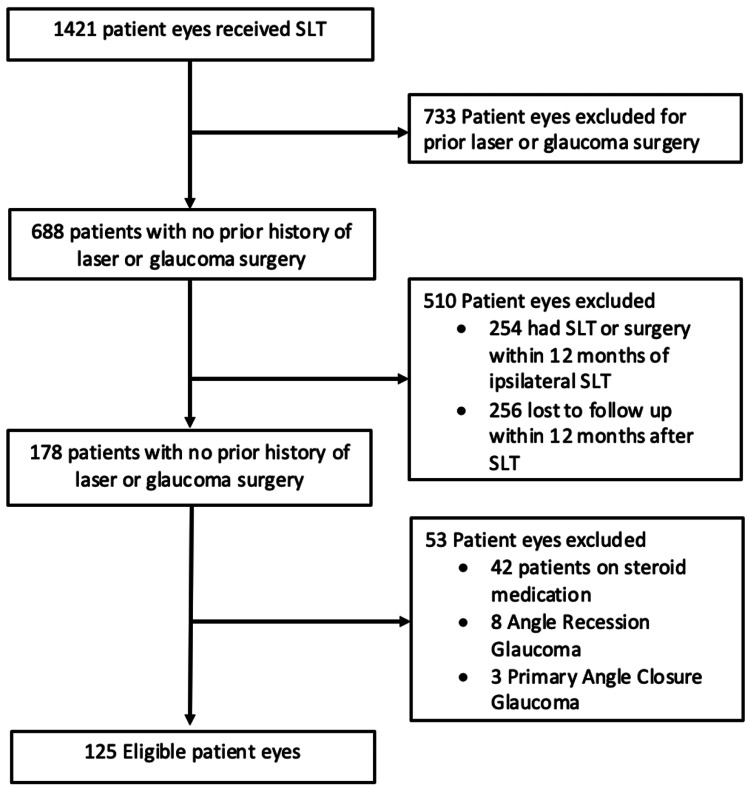
Inclusion and exclusion criteria flowchart

Eyes were excluded from the study if they had a history of previous laser or incisional glaucoma surgeries; underwent additional laser or IOP-lowering surgeries within 12 months before or after SLT; had angle-closure, angle-recession, or combined mechanism glaucoma; or were administered corticosteroids via any route, except for the brief use of topical corticosteroids post-procedure. Inclusion and exclusion criteria are shown in Figure 1.

SLT procedure details

SLT was performed as detailed by Latina et al. [6]. The procedure was carried out by a single experienced surgeon. Following topical anesthetic, each eye received about 100 laser applications for a full 360° of treatment. Energy settings, starting at 0.8 mJ, were adjusted based on trabecular meshwork pigmentation to achieve a visible response. Post-operative care included topical ketorolac medication for two to four days as needed to manage potential inflammation.

Statistical analysis

Collected data included demographic information (such as age, gender, and ethnicity), type of glaucoma, cup-to-disk ratio, central corneal thickness (CCT), IOP, and the number of medications used before and after SLT at six weeks, six months, and 12 months. Success was defined as either achieving a reduction in IOP by at least 20% without requiring additional IOP-lowering medications or procedures including lasers. Continuous variables were analyzed using paired t-tests and ANOVA, while the Wilcoxon rank-sum test was used for non-parametric data. Categorical variables, including SLT success rates, were evaluated with chi-square tests or Fisher’s exact tests as applicable. All statistical analyses were conducted using R (version 4.2.1; R Foundation), with significance set at p<0.05.

## Results

In this study, 125 eyes of 125 patients were analyzed. Demographic and clinical characteristics are presented in Table 1. Notably, the ipsilateral eyes had significantly greater baseline cup-to-disc ratios, IOP, and number of medications.

**Table 1:** Baseline demographic and clinical variables *P-value <0.05 is statistically significant IOP = Intraocular Pressure

Parameter	n = 125	P-value
Age, average years (SD)	65.6 (12.2)	-
Race		-
African-American, n (%)	72 (57.6%)	
Caucasian, n (%)	39 (31.2%)	
Asian, n (%)	7 (5.6%)	
Hispanic/Latino, n (%)	5 (4%)	
Other, n (%)	2 (1.6%)	
Sex		-
Female, n (%)	63 (50.4%)	
Male, n (%)	62 (49.6%)	
Glaucoma type		-
Primary open-angle glaucoma, n (%)	116 (92.8%)	
Normal tension glaucoma, n (%)	4 (3.2%)	
Pigmentary glaucoma, n (%)	3 (2.4%)	
Pseudoexfoliation glaucoma, n (%)	2 (1.6%)	
Vertical C:D ratio, average (SD)		<0.001*
Treated eye	0.76 (0.16)	
Untreated eye	0.66 (0.17)	
Corneal thickness, average (SD)		0.85
Treated eye	535.2 (43.0)	
Untreated eye	535.0 (43.3)	
Baseline IOP, mmHg (SD)		<0.001*
Treated eye	16.63 (4.08)	
Untreated eye	15.21 (3.55)	
Baseline number of medications (SD)	18.7 (6.2)	<0.001*
Treated eye	2.06 (1.29)	
Untreated eye	1.73 (1.29)	

Primary outcomes

**Figure 2:**
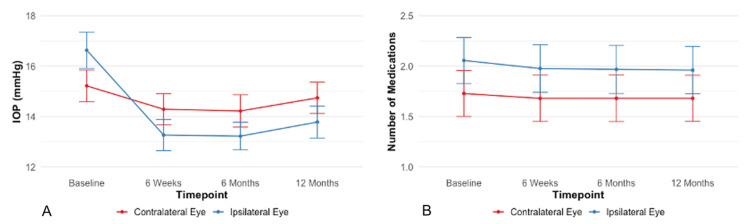
IOP and medication change over time (A) The ipsilateral eye shows sustained IOP reduction, while the contralateral eye's reduction is significant only for up to six months (p<0.05). (B) Both eyes show reduced medication use, with a significant reduction in the contralateral eye only up to six months (p<0.05). Error bars represent standard deviation.

In the contralateral eye, the mean IOP showed a statistically significant reduction at six weeks and six months post-SLT, but not at 12 months (Figure 2A, Table 2). Conversely, the ipsilateral eye exhibited significant IOP reductions at all timepoints. Regarding medication use, the untreated eye had significant reductions at six weeks and six months, but not at 12 months post-SLT (Figure 2B, Table 3). The treated eye, however, experienced significant reductions in medication use at all timepoints.

**Table 2:** IOP reduction over time in the contralateral and ipsilateral eyes *P-value <0.05 is statistically significant. IOP = Intraocular Pressure

		Contralateral Eye			Ipsilateral Eye		
Follow-up Time	Number of Eyes	Mean ± SD (mmHg)	% IOP Reduction	P-value	Mean ± SD (mmHg)	% IOP Reduction	P-value
Post-op 6 weeks	125	14.29 ± 3.50	6.1%	<0.001*	13.26 ± 3.51	20.3%	<0.001*
Post-op 6 months	125	14.22 ± 3.61	6.5%	0.003*	13.22 ± 3.09	20.5%	<0.001*
Post-op 12 months	125	14.74 ± 3.53	3.1%	0.115	13.78 ± 3.59	17.1%	<0.001*

**Table 3:** Medication reduction over time in the contralateral and ipsilateral eyes *P-value <0.05 is statistically significant. Med. = Medication(s)

		Contralateral Eye			Ipsilateral Eye		
Follow-up Time	Number of Eyes	Mean ± SD (Number of Med.)	% Med. Reduction	P-value	Mean ± SD (Number of Med.)	% Med. Reduction	P-value
Post-op 6 weeks	125	1.68 ± 1.30	2.8%	0.033*	1.98 ± 1.33	2.9%	0.001*
Post-op 6 months	125	1.68 ± 1.31	2.8%	0.033*	1.97 ± 1.35	4.3%	0.004*
Post-op 12 months	125	1.68 ± 1.29	2.8%	0.083	1.96 ± 1.33	4.7%	<0.001*

When calculating treatment success rates, the contralateral eye had rates of 24% at both six weeks and six months, which slightly decreased to 20.8% at 12 months. In contrast, the ipsilateral eye showed success rates of 52.8% at six weeks, 49.6% at six months, and remained at 49.6% at the 12-month follow-up. Additionally, the contralateral eye was 5.05 times more likely to be successful if the ipsilateral eye was successful at six weeks (odds ratio (95% confidence interval): 5.05 (1.89-13.48)), 16.1 times more likely at six months (16.1 (4.56-57.17)), and 5.94 times more likely at 12 months (5.94 (2.07-17.04)) (p<0.001 for all timepoints) (Table 4).

**Table 4:** Success comparison between contralateral and ipsilateral eyes at follow-up intervals *P-value <0.05 is statistically significant. CI = 95% Confidence Interval

Parameters		
6 Weeks	Contralateral Eye Success	Contralateral Eye Failure
Ipsilateral Eye Success	24	42
Ipsilateral Eye Failure	6	53
P-value	< 0.001*	
Odds ratio [CI]	5.05 CI [1.89 - 13.48]	
6 Months	Contralateral Eye Success	Contralateral Eye Failure
Ipsilateral Eye Success	27	34
Ipsilateral Eye Failure	3	61
P-value	< 0.001*	
Odds ratio [CI]	16.1 [4.56-57.17]	
12 Months	Contralateral Eye Success	Contralateral Eye Failure
Ipsilateral Eye Success	21	41
Ipsilateral Eye Failure	5	58
P-value	< 0.001*	
Odds ratio [CI]	5.94 [2.07-17.04]	

## Discussion

This study aimed to investigate the impact of SLT on IOP and medication reduction in the contralateral eye and to evaluate whether success in the ipsilateral eye influences contralateral eye outcomes. Our findings show that SLT significantly reduces IOP and medication use in the untreated eye for up to six months following treatment. Additionally, the untreated eye was 5.05-16.1 times more likely to achieve success if the treated eye was successful within 12 months of follow-up.

Our cohort demonstrated a similar or lesser IOP reduction in the contralateral eye compared to prior reports [6,7,9]. Compared to our cohort, studies reporting a higher reduction of IOP in the contralateral eye have also reported higher baseline IOPs. This trend may explain the disparity in our results as research has shown that higher baseline IOP is correlated with a larger amplitude in IOP reduction following SLT [7,9]. Additionally, we observed a statistically significant reduction in the mean number of medications in the untreated eye post-SLT. Although previous reports have shown that SLT can replace or decrease the number of medications in the treated eye, to our knowledge, this is the first report showing a statistically significant decrease in the number of medications in the contralateral eye [13-18]. However, this reduction is clinically insignificant and does not serve as a basis to change glaucoma management preemptively.

Furthermore, it is worth noting that the effect of SLT in the contralateral eye is most pronounced within the first six months of treatment. Our study demonstrated a treatment success rate of 24.0% at both six weeks and six months, decreasing to 20.8% at 12 months in the untreated eye. While there have been similar success rates in other studies (e.g., Liu et al. reported 24.0% at six months and 22.2% at 12 months [9], Hirabayashi et al. observed 36.8% at six months [11]), their success criteria either did not account for increases in medication [9] or included subjective criteria that may vary among providers (≥1 decrease in medications) [11], limiting their results. Additionally, we found that untreated eyes are five to 16 times more likely to respond successfully to treatment if the treated eye achieved success (Table 4). This finding builds on previous research showing that contralateral eyes were six times more likely to be successful if the treated eye was successful at six months and contributes to our understanding of the dual impact of SLT [11].

This study’s population improves on the previous study’s external validity. Our study has the greatest sample size (n=125), diminishing the effect of outliers on our mean IOP compared to other studies (previously 85 [10], 74 [11], 53 [6], 43 [7], 32 [9], and 20 patients [8]). Moreover, this work comprised 125 patients of diverse backgrounds (African American (57.6%), Caucasian (31.2%), Asian (5.6%), Hispanic/Latino (4%), and not reported (1.6%)). Only Latina et al. reported the effects of SLT in the contralateral eye in a heterogenous population [6], while backgrounds in other studies were either unreported [7,12] or nondiverse [8-11].

The mechanism by which SLT reduces IOP of the contralateral eye has yet to be elucidated. One hypothesis suggests that the thermal energy from unilateral SLT produces a systemic increase in cytokine secretion, matrix metalloproteinase induction, increase in cell division, and macrophage recruitment that acts on the trabecular meshwork of both eyes to increase aqueous outflow and decrease IOP [19]. Evidence of contralateral pressure changes in SLT, as well as in other procedures such as trabeculectomy, supports an IOP-modulating role in postoperative cytokine secretion, not limited to decreases in IOP [20,21]. Our findings of a statistically significant reduction in IOP and medications over six months, along with the increased likelihood of success in the untreated eye when the treated eye is successful, further support the hypothesis that systemic mediators are at work. Further studies are needed to fully elucidate the mechanism.

Limitations of this study include its retrospective design and small sample size. This retrospective design lacked a control group and had a smaller sample size largely due to the exclusion criteria of having prior laser treatment. Due to the retrospective design, there is a potential for overestimation of the IOP lower effect of SLT as the preoperative IOP is considered the baseline. The current study was also unable to control for the effect of increased medication compliance following SLT. Additionally, the use of a single provider's clinical judgment for medication adjustments could lead to a skew in success rates due to the lack of variability in treatment approaches that might otherwise be present with multiple providers. Furthermore, the exclusion of patients who needed repeat SLT or incisional surgery within 12 months after SLT due to a lack of response leads to an overestimation of the effect of SLT. Future studies on this topic should include a large, multi-surgeon prospective clinical trial with controls for medication compliance.

## Conclusions

First-time SLT leads to statistically significant but not clinically significant reductions in IOP and medication use in the contralateral eye for up to six months post treatment. Additionally, achieving a successful outcome in the ipsilateral eye significantly predicts a favorable response in the contralateral eye within one year of treatment. Future prospective studies with larger sample sizes and controlled variables are essential to further elucidate the mechanisms behind SLT’s systemic effects and to validate these findings.
